# Supplementary material for: Multi-Chemical Omics Analysis of the Symbiodiniaceae Durusdinium trenchii under Heat Stress
Source: Microorganisms. 2024 Feb 2;12(2):317. doi: 10.3390/microorganisms12020317 (PMC10893086; doi:10.3390/microorganisms12020317)
Supplement: Supplementary file 1 [file microorganisms-12-00317-s001.zip › Supplementary_Material_Matthews et al_resub.pdf]

Supplementary Material for

Multi-chemical omics analysis of the Symbiodiniaceae *Durusdinium trenchii* under heat-stress

Jennifer L. Matthews[1*], Maiken Ueland[2,3], Caitlin A. Lawson[1], Natasha Bartels[1], Thomas Lockwood[3], Yida Wu[1], Emma F. Camp[1*]

*** Correspondence:** Corresponding Authors:

Jennifer.matthews@uts.edu.au, emma.camp@uts.edu.au

Supplementary Figures

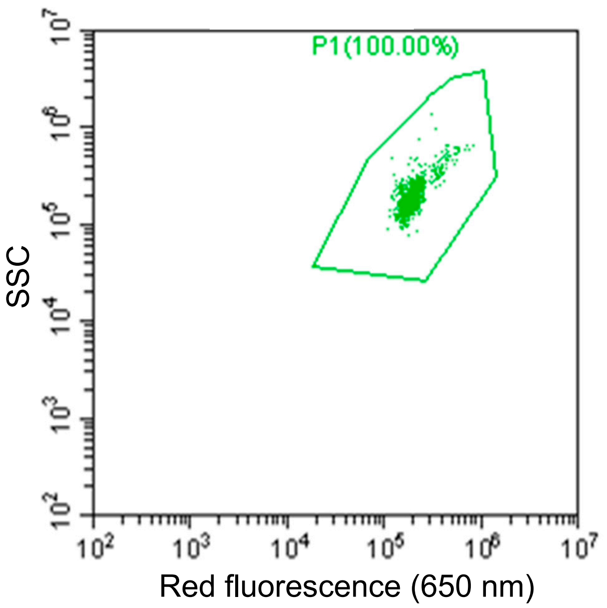

Figure S1. Gating strategy used to collect flow cytometry data. Symbiodiniaceae population characterised according to side scatter (SSC) and red fluorescence (chlorophyll).
